# Neural Synchrony and Network Dynamics in Social Interaction: A Hyper-Brain Cell Assembly Hypothesis

**DOI:** 10.3389/fnhum.2022.848026

**Published:** 2022-04-29

**Authors:** Viktor Müller

**Affiliations:** Center for Lifespan Psychology, Max Planck Institute for Human Development, Berlin, Germany

**Keywords:** neural synchrony, hyper-brain network dynamics, within- and cross-frequency coupling, multiplex and multilayer networks, graph-theoretic approach, social interaction

## Abstract

Mounting neurophysiological evidence suggests that interpersonal interaction relies on continual communication between cell assemblies within interacting brains and continual adjustments of these neuronal dynamic states between the brains. In this Hypothesis and Theory article, a *Hyper-Brain Cell Assembly Hypothesis* is suggested on the basis of a conceptual review of neural synchrony and network dynamics and their roles in emerging cell assemblies within the interacting brains. The proposed hypothesis states that such cell assemblies can emerge not only within, but also between the interacting brains. More precisely, the *hyper-brain cell assembly* encompasses and integrates oscillatory activity within and between brains, and represents a common hyper-brain unit, which has a certain relation to social behavior and interaction. Hyper-brain modules or communities, comprising nodes across two or several brains, are considered as one of the possible representations of the hypothesized *hyper-brain cell assemblies*, which can also have a multidimensional or multilayer structure. It is concluded that the neuronal dynamics during interpersonal interaction is brain-wide, i.e., it is based on common neuronal activity of several brains or, more generally, of the coupled physiological systems including brains.

## Introduction

As noted by György Buzsáki (2006, p. 5), “Nature is both periodic and perpetual. One of the most basic laws of the universe is the law of periodicity. This law governs all manifestations of living and nonliving. In its broadest definition, periodicity refers to the quality, state, or fact of being regularly recurrent: a repeating pattern or structure in time or space. What goes up must come down. The sun rises and sets, and the days wax and wane. Without periodicity, there is no time; without time, there is no past, present, or future. In living systems, the periodicity of individual lives gives rise to the continuity of life on Earth. Our existence has meaning only when experienced in time.” The most common expression of periodicity is an oscillation. As indicated in several studies, most biological, and also social systems, are oscillatory in nature (Stankovski et al., 2015; Cao et al., 2016; Wilson and Cook, 2016; Müller et al., 2018a). Oscillations form a basis for communication between different components or subsystems at different organizational levels and between them. In line with synergetics and a system dynamics approach, complex natural phenomena can best be described by the interaction of several subsystems (Forrester, 1968; Haken’s, 1983; Haken, 1984). When viewed in isolation, each of these subsystems has its own (often relatively simple) dynamics; however, the complex behavior of the entire system is due to corresponding coupling mechanisms within and between these subsystems that can be considered as a superordinate system imposing boundary conditions on its constituents (Buzsáki, 2006; Haken, 2006, 2016; Noble, 2012; Haken and Portugali, 2016, 2021; Müller et al., 2018a, 2019a, 2021a). As noted by Arkady Pikovsky and colleagues (Pikovsky et al., 2003, pp. xvii–xviii), “Our surroundings are full of oscillating objects. Radio communication and electrical equipment, violins in an orchestra, fireflies emitting sequences of light pulses, crickets producing chirps, birds flapping their wings, chemical systems exhibiting oscillatory variation of the concentration of reagents, a neural center that controls the contraction of the human heart and the heart itself, a center of pathological activity that causes involuntary shaking of limbs as a consequence of Parkinson’s disease—all these and many other systems have a common feature: they produce rhythms. Usually, these objects are not isolated from their environment, but interact with other objects, in other words, they are open systems.” As the authors then concluded: “**This adjustment of rhythms due to an interaction is the essence of synchronization**…” (Pikovsky et al., 2003, p. xviii).

Such relations between things or systems are also crucial for our social life. In everyday life, people often have to coordinate their actions with each other in time and space. Such interpersonal interaction is also essential for the development of congruent goals, which are generally decisive for social behavior, although not always necessary for the mutual alignment of minds and bodies (Gallotti and Frith, 2013; Gallotti et al., 2017; Müller et al., 2021a). As proposed by Hasson and Frith (2016, p. 2), “…interactions with other members of a group can fundamentally shape the way we behave in the world, and alignment is a ubiquitous feature of such interactions.” Such an alignment presupposes different types of synchronization and/or temporal (and also spatial) coordination.

In biological studies of collective behavior, interactions among individual insects, and between them and their environment, result in swarm behavior that is often considered a “superorganism” (Trianni et al., 2011). The concept of superorganism was proposed by the American entomologist William Morton Wheeler (1911) in his seminal work “The ant-colony as an organism” (cf. Hoffecker, 2013). The ability to function as a whole and to take collective decisions is also a representative feature of other organisms or societies (e.g., fish schools, bird flocks, packs or herds of mammals) as well as human group behavior (Detrain and Deneubourg, 2006; Gelblum et al., 2015; Müller et al., 2021a). Such societies are self-organizing and auto-regulating systems. Self-organization is defined as a process where a spontaneous order or a spatiotemporal pattern arises from local interactions between parts of an initially disordered system, mostly triggered by random fluctuations and amplified by positive feedback. The self-organizing pattern of order is an emergent property of the system that arises unexpectedly from interactions among the system’s components (Haken’s, 1983; Haken, 1984, 2006; Haken and Portugali, 2016, 2021). As noted by Ilya Prigogine and colleagues, self-organization typically takes place in non-linear systems, which are far from their thermodynamic equilibrium state (Nicolis and Prigogine, 1977; Prigogine and Stengers, 1984). Ross Ashby (1952) also suggested that a dynamical system (far from an equilibrium state) always tends to evolve towards a stable equilibrium state or towards an attractor in a phase space.

The present article aims at providing a conceptual review of neural synchrony and network dynamics in social interaction. First, I will review synchronization phenomena in general and how they emerge in social interaction and interpersonal action coordination in particular. Second, different types of neural coding (e.g., rate and temporal codes) and their roles in emerging cell assemblies will be discussed in relation to brain oscillations and underlying coupling dynamics. Third, after considering the intra- and inter-brain synchronization processes with a reference to hyper-brain activity, a *Hyper-Brain Cell Assembly Hypothesis* will be postulated or suggested. Fourth, neural and hyper-brain network dynamics will be considered in terms of cross-frequency coupling and multiplex or multilayer networks. Hyper-brain modules or communities will be considered as one of the possible representations of hypothesized *hyper-brain cell assemblies*, which can also have a multilayer or multidimensional structure. Fifth, network physiology and neural network dynamics will be inspected in their unified interplay. Investigating the hyper-brain networks in their close connections with physiological systems and subsystems is crucial for understanding the mechanisms of social interaction and interpersonal action coordination. Finally, ways of proving the hyper-brain cell assembly hypothesis will be provided and discussed.

## Synchronization Phenomena and Their Role in Social Interaction

The German biologist and physiologist Erich von Holst arguably made the most important contribution to the topic of synchronization and coordination. His fundamental work established the concepts of absolute and relative coordination drawing on the example of motion sequences in animals (von Holst, 1936a,b, 1938a,b, 1939a,b). The generic term of fixed or *absolute coordination* denotes all movement types in which the frequency and amplitude of a movement remain constant and the individual limbs or segments maintain a certain phase relationship characteristic of the form of coordination in question. Its counterpart, sliding or *relative coordination*, is fundamentally different from absolute coordination, as the characteristics of constancy of frequency, amplitude, and/or phase relations do not apply. In relative coordination, the different moving parts (at least some of them) have a different working cycle and correspondingly changing mutual phase relations; in addition, one or the other rhythm usually shows a more or less distinct periodic change of amplitude and frequency. Formally this largely corresponds to the periodic forms of harmonic or quasi-harmonic oscillations (cf. von Holst, 1939a, b). The so-called “magnet effect” was considered to be the central mechanism of relative coordination (von Holst, 1936b). It states that two (or more) rhythms can influence each other in different ways. These interactions can show different behavior that mostly depends on the strength of one or the other rhythm. If, for example, the effect is only transferred from one rhythm to the other, the one exerting the effect while remaining uninfluenced itself is described as attracting and the other as attracted. If the effect is very strong, it can ultimately lead to absolute coordination, with the attracted rhythm completely taking over the oscillation frequency of the attracting rhythm. If both rhythms influence each other, one speaks of a mutual attraction. Normally, a mutual attraction slows down the faster rhythm and accelerates the slower one (von Holst, 1936b, 1938a,b, 1939a,b). As Erich von Holst has shown, the frequency relationships are not “rigid” and change continually. A comparably simple model for frequency adaptation of populations of coupled oscillators was proposed by Ott and Antonsen (2017). Despite the fact that von Holst’s main ideas about absolute and relative synchronization were derived from the motion sequences in animals, they are highly transferable to neuronal and other (e.g., social) processes.

A further interesting point about oscillatory synchrony was made by György Buzsáki (2006): “The paramount advantage of synchronization by oscillation is its cost-effectiveness. No other known mechanism in the physical world can bring about synchrony with so little investment. What do I mean by declaring that synchrony by oscillation is cheap? Let me illustrate the cost issue first with a few familiar examples from our everyday life. You have probably watched leisurely strolling romantic couples on a fine evening in a park or on the beach. Couples holding hands walk in perfect unison, whereas couples without such physical links walk out of step” (Buzsáki, 2006, p. 168). Cost-effectiveness is probably the most important factor that made oscillatory synchrony ubiquitous. As suggested by Karl Friston and colleagues, oscillatory synchrony is an emergent property of free energy minimization (Friston, 2010; Friston and Frith, 2015; Palacios et al., 2019). A further familiar example from our everyday life is synchronized clapping or applause, which provides a striking example of social self-organization (Néda et al., 2000a, b). Néda et al. (2000a) describe it as follows: “Our measurements offer an insight into the mechanism of synchronized clapping: during fast clapping, synchronization is not possible owing to the large dispersion in the clapping frequencies. After period doubling, as mode II clapping with small dispersion appears, synchronization can be and is achieved. However, as the audience gradually decreases the period to enhance the average noise intensity, it slips back to the fast clapping mode with larger dispersion, destroying synchronization” (Néda et al., 2000a, p. 849). Thus, the two main desires and intentions of the spectators (i.e., optimal synchronization and maximal applause intensity) cannot both be fulfilled at the same time (Néda et al., 2000b). It is obvious that synchronized clapping saves us costs while applause intensity is more costly. In accordance with the free-energy principle, any self-organizing system that is at equilibrium with its environment must minimize its free energy (Friston et al., 2006; Friston, 2010; Bruineberg et al., 2018; Palacios et al., 2019). Moreover, this switching between maximal applause intensity and optimal synchronization is also a good example of phase transition and the emergence of order from chaos or enhanced fluctuations (cf. Prigogine and Stengers, 1984; Haken, 2006; Haken and Portugali, 2016, 2021). It also shows the way self-organization may occur.

Another interesting aspect of the applause scenario is that synchronous clapping is also rhythmic. For a short moment, there is competition among the different individual rhythms, but very quickly people “agree” on a certain rhythm that is compatible with their state and corresponds to the specific situation (Kriz, 2001). In everyday life, rhythm plays an important, often even a central role, not only for applause or such rhythmic behavior as music, speech, or dancing (Brown, 2018; Kotz et al., 2018; Savage et al., 2021), but also in architecture, poetry, cinema, theater, arts, and many other domains of human life (Chan, 2012; Cureton, 2015; Thapa, 2017; Mohamed, 2018). Rhythm is omnipresent and represents a basic anthropological principle that determines the bodily-sensory experience of space and time and is considered a fundamental *organizing principle* of social interaction (Iberall and McCulloch, 1968, 1969; Warner et al., 1987; Warner, 1988).

## Neural Coding, Cell Assemblies, Brain Oscillations, and Coupling Dynamics

### Neural Coding and Oscillatory Activity

From the neural point of view, oscillations reflect rhythmic synchronous firing of neuronal elements or cells. Adrian (1926a,b); Adrian and Zotterman (1926a,b); and Adrian and Bronk (1928, 1929) showed that the intensity or salience of a stimulus is often coded as the rate of nerve action potentials over time. This is commonly referred to as the “rate code” (Gerstner et al., 1997; Mehta et al., 2002; Panzeri et al., 2015; Li and Tsien, 2017). An alternative coding concept, known as “temporal coding,” considers the exact timing of neural spikes and is also relevant for the coding of sensory and motor events (Gerstner et al., 1997; Mehta et al., 2002; Li and Tsien, 2017). Temporal coding becomes more robust with experience and drives synaptic plasticity in cortical networks (Mehta et al., 2002; Hestrin and Galarreta, 2005). It has been shown that the rate and temporal codes can be highly correlated (Mehta et al., 2002) or also independently adjustable (Singer, 1999). As suggested by Singer (1999, p. 56), “…fast synchronization codes and more sustained rate codes could ultimately coexist and perhaps even optimally complement one another.” Thus, internal or external information is encoded by not only modulating the firing rate of individual neurons but also by temporally synchronized spiking across different neurons. They mostly participate in synchronously oscillating assemblies and give rise to the robust real-time population or cell-assembly code (Singer, 1999; Li and Tsien, 2017). A candidate mechanism for representation of neural coding at the macroscopic level is oscillatory brain activity such as hippocampal theta or gamma oscillations (Buzsáki and Draguhn, 2004; Siapas et al., 2005) or cortical and thalamocortical alpha oscillatory activity (Buzsáki, 2006; Klimesch, 2012; Becker et al., 2015).

The basic characteristics of an oscillation are its amplitude, phase, and frequency, changing across time. These changes are a result of different neural coding strategies that complement each other. If the amplitude of the signal is coded by the firing rate or “rate code,” its temporal structure or phase is instead reflected by the “temporal code”. The frequency of an oscillation is an indicator of change or rate of change, i.e., it shows how many cycles per second occur. Important characteristics of neural coding mechanisms are synchronization, in general, and phase synchronization among temporally linked processes, in particular (Llinás et al., 2005).

The electroencephalogram (EEG) offers a rich source of information about neural coding dynamics (Nunez’s, 1995; Nunez, 2000). Neuronal oscillations in mammalian cortical networks can be observed across a wide frequency range from approximately 0.05 to 500 Hz and have been regarded as a key mechanism in neural communication and cognitive brain function (Buzsáki and Draguhn, 2004; Buzsáki, 2006). Enhanced oscillatory activity in the delta frequency range (0.05–3.5 Hz) during cognitive tasks is often considered an indicator of attentional task demands (Harmony et al., 1996, 2001; McEvoy et al., 2001). In contrast, in the absence of cognitive tasks, delta oscillations have been associated with slow-wave sleep and anesthesia (Blake and Gerard, 1937; Chauvette et al., 2011; Nir et al., 2011; Hagihira, 2015). In addition, phase-amplitude coupling between delta and high-frequency oscillations was observed during sub-anesthetic and general anesthetic sevoflurane treated brain states; thus, delta brain oscillations may modulate high-frequency brain activity in respective manners (Chamadia et al., 2019). Furthermore, coherent large-scale delta oscillations play a crucial role during decision-making (Nácher et al., 2013). Theta oscillations (4–7 Hz) are particularly prominent, with possible functional roles covering a wide range of behavior from arousal, attention, and memory to the orienting reflex, conditioning, and learning, including different binding and information processing mechanisms as well as large-scale integrative processes (Buzsáki, 2005, 2006; Müller et al., 2009). In contrast to delta and theta frequencies, alpha (8–13 Hz) and partly also beta (14–28 Hz) rhythms tend to respond to a stimulus and/or task demands either with a decrease or increase in amplitude/power, termed event-related desynchronization and synchronization, respectively (Klimesch, 2012). These rhythms decrease or desynchronize, for example, in response to the perceptual decision and memory tasks (Pfurtscheller and Lopes da Silva, 1999). At the same time, brain regions associated with task-irrelevant and potentially interfering processes exhibit event-related synchronization, especially in the alpha band, emphasizing the inhibitory function of alpha oscillations (Pfurtscheller and Lopes da Silva, 1999; Klimesch, 2012). However, it has also been suggested that local and inter-areal alpha phase synchronization may play a role in active task-relevant neuronal processing and support attentional, executive, contextual functions, and consciousness (Palva and Palva, 2007, 2011). Moreover, alpha oscillations can be synchronized in terms of cross-frequency coupling with theta, beta, and gamma oscillations in response to cognitive demand, attention, and sensory awareness (Palva et al., 2005; Schack et al., 2005; Palva and Palva, 2007). Gamma band oscillations (>30 Hz) have been demonstrated to play an important role in perception, perceptual binding phenomena, memory, and synaptic plasticity (Tallon-Baudry and Bertrand, 1999; Singer, 2001; Fries et al., 2007; Van Vugt et al., 2010; Zarnadze et al., 2016; Bocchio et al., 2017; von Lautz et al., 2017; Galuske et al., 2019). In cortical networks, a dynamic balance between excitation and inhibition leads to a series of network oscillations involving neuronal populations of different sizes (Buzsáki, 2007). In general, network oscillations may functionally bias stimulus selection, support transient bindings of neuronal assemblies, and facilitate synaptic plasticity (Bibbig et al., 2001; Fries et al., 2007; Zarnadze et al., 2016; Bocchio et al., 2017; Galuske et al., 2019).

Varela et al. (2001) distinguished between local synchrony (on a spatial scale of less than 1 cm and conduction delay of 4–6 ms) and global or large-scale synchrony (on a spatial scale of more than 1 cm and conduction delay of 8–10 ms) providing neural integration on different organization levels. In agreement with Nunez’s (1995) dynamic theory, it will be distinguished between local processes (with a dominance of functional segregation) and global ones (with a dominance of functional integration) that are in steady interaction (Nunez’s, 1995; Nunez, 2000). In line with the dynamic core hypothesis proposed by Tononi and Edelman (1998, p. 1848), “…the distributed neural process underlying conscious experience must be functionally integrated and at the same time highly differentiated.” Neural processes with high functional segregation and integration reveal high neural complexity (in terms of the mutual information between subsets of the system’s units). However, complexity can also be low when the components of a system are either completely independent (segregated) or completely dependent (integrated). It has been shown that certain structural characteristics of the brain, such as a high density of connections, strong local connectivity, patchiness in the connectivity among neuronal groups, and a large number of reentrant circuits, are associated with high neural complexity and conscious experiences (Tononi et al., 1994; Tononi and Edelman, 1998). Buzsáki (2007) also suggested that the local-global relations of the cerebral cortex and the ongoing, self-organized complex dynamics are necessary ingredients for subjective experiences. He argued that understanding and distinguishing between normal and dysfunctional processes in the cerebral cortex can be enabled by linking local and global patterns of activity on behaviorally relevant time scales. In line with Haken’s (1983) principles of synergetics, “…emergence through self-organization has two directions. The upward direction is the local-to-global causation, through which novel dynamics emerge. The downward direction is a global-to-local determination, whereby a global order parameter “enslaves” the constituents and effectively governs local interactions. There is no supervisor or agent that causes order; the system is self-organized. The spooky thing here, of course, is that while the parts do cause the behavior of the whole, the behavior of the whole also constrains the behavior of its parts according to a majority rule; it is a case of circular causation. Crucially, the cause is not one or the other but is embedded in the configuration of relations” (Buzsáki, 2006, p. 14; see also Haken’s, 1983; Haken, 2006).

### The Neural Cell Assembly Approach

Self-organization is also an important element in the cell assembly approach or Hebbian learning (Hebb, 1949). In the early 1920s, Karl Lashley began his historical work on memory traces (engrams) in the cerebral cortex. He showed that distribution of active and inactive synapses provided evidence for learning or memory processes (Lashley, 1924, 1931). Lashley’s student, Donald Hebb, developed his so-called theory of cell assemblies, which he published in 1949 in his well-known book, *The Organization of Behavior*. He postulated: “*When an axon of cell* A* is near enough to excite a cell* B* and repeatedly or persistently takes part in firing it, some growth process or metabolic change takes place in one or both cells such that* A*’s efficiency, as one of the cells firing* B*, is increased*” (Hebb, 1949, p. 62). Hebb (1949) used the concept of “reverberating circuits” or “closed self-reexciting chains”, proposed by Lorente de Nó (1933, 1938) to describe a mechanism for maintaining activity in the cortex after the initial stimulus has ceased. This led him to the concept of the *cell assembly*, a complex reverberating circuit that can be created during an experience/behavior by certain changes in the synapses. Once activated, such a circuit can maintain excitation in the neural system, whereby each neuron in the cell assembly receives excitation from and transmits it to other neurons of the same assembly. When most of the neuronal elements in such a cell assembly become active, the whole cell assembly fires, i.e., the cell assembly represents an entity (according to the Hebbian rule: “what fires together, wires together”). The formation and development of cell assemblies are closely related to plastic (“Hebbian”) excitatory cell systems with a rapid activation time constant. The velocity component is very important because the cell assemblies must have the property to ignite explosively as a whole. As mentioned above, a dynamic balance between excitation and inhibition is important to ensure that information from excitatory cells controlled by inhibitory interneurons flows to just the right place at just the right time (Buzsáki, 2007). Thus, a cell assembly forms a functional unit within the entire neuronal network and can provide a neural representation of objects, concepts, thoughts, and various behavioral patterns (cf. Birbaumer et al., 1995; Müller et al., 2011). As figuratively expressed by Pulvermüller et al. (2014, p. 575) “…cell assemblies may be the neurobiological vehicles of perception, action, attention, memory, decision, concepts, language and thought…”. Moreover, as stated by Buzsáki: “…flexible cooperation among local and distant cell assemblies is believed to underlie nearly all cognitive behaviors” (Buzsáki, 2006, p. 48). Buzsáki also discussed the importance of neuronal synchronization in the formation of functional cell assemblies and concluded that “synchronization by oscillation is the simplest and most economic mechanism to bring together discharging neurons in time so that they can exert a maximal impact on their targets” (Buzsáki, 2006, p. 137). Cell assemblies active within an oscillation cycle can represent an integrated entity (e.g., a neural “letter”) and the chaining of such assemblies (Hebb’s “phase sequences”) would provide the basis for complex cognitive processes (e.g., neural “words”). Hence, there is an intrinsic relation between oscillatory activity, neural cell assemblies, and behavioral or cognitive entities (Buzsáki, 2006, 2010). As suggested by Varela et al. (2001, p. 229), “… the emergence of a specific neuronal assembly is thought to underlie the operation of every cognitive act.”

## Intra- and Inter-Brain Synchronization and The Hyper-Brain Cell Assembly Hypothesis

### Intra- and Inter-Brain Synchronization as a Hyper-Brain Activity

A recently emerging view in cognitive neuroscience with regard to hyperscanning methods holds that interpersonal action coordination or social interaction (e.g., playing music in duets or groups, dancing, acrobatics, competitive sports, movement imitation, etc.) requires strong inter-brain synchronization (synchronized neuronal activities in multiple brains) and specific hyper-brain network activity to support such coordination or interaction (Lindenberger et al., 2009; Astolfi et al., 2010, 2020; Dumas et al., 2010, 2012, 2020; Sänger et al., 2012, 2013, 2013; Müller et al., 2018b; Acquadro et al., 2016; Müller and Lindenberger, 2019, 2022; Czeszumski et al., 2020). Synchronization both within and between brains seems to be crucial for interpersonal interaction and is an inevitable element of neuronal communication systems within and between individuals or agents (Sänger et al., 2011, 2012, 2013; Yun et al., 2012; Dumas et al., 2012; Müller et al., 2013, 2018b, 2021a; Müller and Lindenberger, 2014, 2019, 2022; Szymanski et al., 2017b; Hu et al., 2017; Ahn et al., 2018; Stone et al., 2019; Astolfi et al., 2020; Balconi et al., 2020). In our view, both these forms of synchronization (i.e., intra- and inter-brain synchronization), reflecting the common integrated state of interactors known as *hyper-brain* *network* activity, are of paramount importance. This hyper-brain network activity is enhanced during periods of high demand on interpersonal interaction, and exhibits temporal and structural changes in response to the current situation and social circumstances (Sänger et al., 2012, 2013; Yun et al., 2012; Müller et al., 2013, 2018b; Filho et al., 2016; Toppi et al., 2016; Müller and Lindenberger, 2019, 2022; Astolfi et al., 2020). In an EEG hyperscanning study on fingertip movement, it has been shown that the overall number of significant phase synchrony in theta and beta frequency bands increased after cooperative interaction training in inter-brain connections, but not in intra-brain connections (Yun et al., 2012). In a computerized joint action task including interactive and non-interactive conditions, hyper-brain EEG activity was investigated using graph-theoretical approach (GTA) measures (Astolfi et al., 2020). Results of this study indicated that all the GTA indices were modulated by the interaction task, and returned a significantly stronger integration of hyper-brain networks in the interactive vs. non-interactive conditions. The authors compared also GTA indices derived from hyper-brain and intra-brain networks and showed that the former differentiated better between the conditions at lower frequencies (theta and alpha) and the latter differentiated better at higher frequencies (beta and gamma). The hyper-brain GTA indices were also modulated in accordance with the degree of cooperation or successful interaction between subjects (Astolfi et al., 2020). Filho et al. (2016) investigated within- and between-brain connectivity during a dyadic juggling task. Although the study was carried out with only one dyad of jugglers, the data showed some interesting synchronization patterns both within and between brains as well as corresponding changes in hyper-brain network dynamics in the theta and alpha frequency bands, which vary as a function of task difficulty (i.e., three, four, five, or six balls juggled). In another study with 13 jugglers divided into seven pairs (one juggler participated in two different pairs), Stone et al. (2019) found that at the intra-brain level, global efficiency was reduced for less-skilled jugglers and increased for more skilled jugglers during paired juggling as compared to solo juggling. No significant results were found at the inter- or hyper-brain levels in this study (Stone et al., 2019). Within- and between-brain connectivity, as well as the corresponding hyper-brain network structure and topology dynamics, were found to change as a function of playing condition, musical situation, and musical role (e.g., leader vs. follower) as well as the oscillation frequency in guitarist duets and quartets (Sänger et al., 2012, 2013; Müller et al., 2013, 2018b; Müller and Lindenberger, 2019, 2022). However, there is a large gap between neural and behavioral data—we neither know exactly how intra- and inter-brain synchronous activity influence or promote each other and how this interaction, resulting in hyper-brain network activity, is related to social behavior nor whether and to what degree internal state variables contribute to inter-brain dynamics (cf. Kingsbury and Hong, 2020). Recently, it has been suggested that within-brain activity and connectivity reflect an individual state of the interacting agent, while the adjustment of these activity states during interpersonal interaction can only be facilitated by between-brain connectivity or synchrony (Müller et al., 2021a). Thus, interpersonal interaction is locked into steady communication between the cell assemblies within the interacting brains and continual adjustment of these dynamic neuronal states between the brains (Müller et al., 2021a; Shamay-Tsoory, 2021).

**Figure 1:**
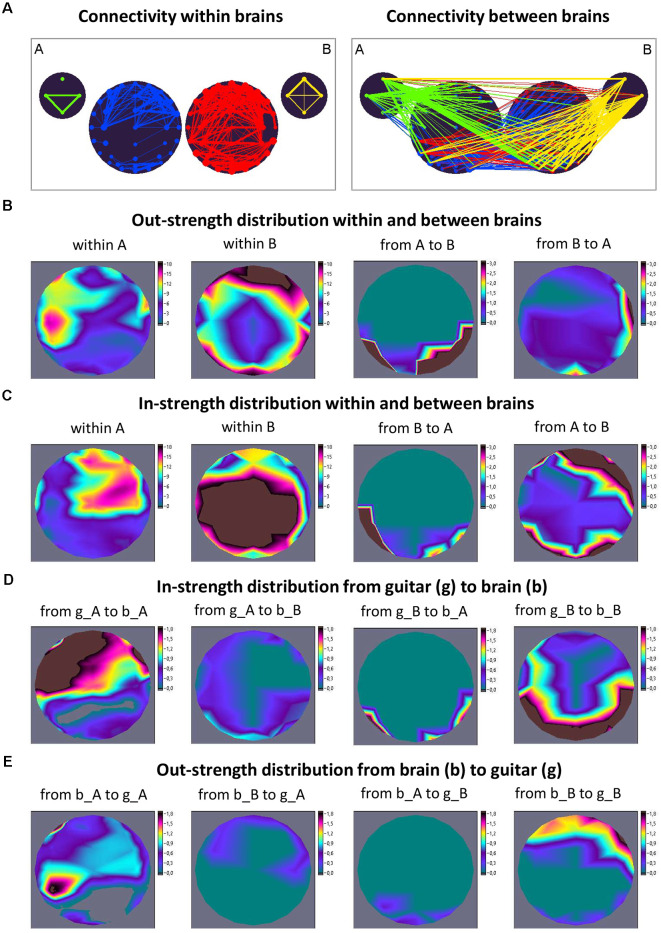
Connectivity within and between the brains and instruments and corresponding strength distributions during free guitar improvisation.** (A)** Connectivity maps. The left panel represents the connectivity between the guitars and brains, and the right panel represents the connectivity between the guitars and brains. The strength of the nodes (sum of all out-going connections) is coded by circle size, and the strength of edges is coded by line thickness. The different parts of the network are color-coded: guitar A, green; guitar B, yellow; guitarist A’s brain, blue; guitarist B’s brain, red. The guitars comprise four nodes each, indicating different frequency ranges of auditory signals ordered clockwise from top: low, middle, high, and whole range (see text). The brains comprise 40 nodes (electrodes) each. **(B)** Out-strength distribution within and between brains. The two maps on the left represent the topological distribution of the out-strengths within the brains of the two guitarists, and the ones on the right display the topological distribution of the out-strengths going from guitarist A’s brain to guitarist B’s brain and *vice versa*. **(C)** In-strength distribution within and between brains. The two maps on the left represent the topological distribution of the in-strengths within the brains of the two guitarists, and the ones on the right display the topological distribution of the in-strengths coming from guitarist B’s brain to guitarist A’s brain and* vice versa*. **(D)** In-strength distribution from the guitar (g) to the brain (b). The strength distribution maps from left to right represent the topological distribution of the in-strengths coming from guitar A to brain A, from guitar A to brain B, from guitar B to brain A, and from guitar B to brain B. **(E)** Out-strength distribution from the brain (b) to guitar (g). The strength distribution maps from left to right represent the topological distribution of the out-strengths coming from brain A to guitar A, from brain B to guitar A, from brain A to guitar B, and from brain B to guitar B. It can be seen that the guitars address brain regions in the two brains that are interconnected as well as those that are not. In addition, there are connections between the brains that are not connected with the guitars (adapted from Müller and Lindenberger, 2019).

However, we have to keep in mind that there is an essential difference between intra- and inter-brain synchronization. While synchronization within the brain is mostly (but not always) bound to the neuronal substrate, synchronization between brains is substrate-free (in the sense that there are no physical connections between the brains) and presumably relies on the common timing of the interactors. It has also been suggested that inter-brain synchronization could be a result of shared perceptual input and/or equal motor output, but nevertheless, there is evidence that a certain amount of inter-brain synchronization has intrinsic attraction and is not necessarily (directly) caused by common systems’ input or output (Lindenberger et al., 2009; Sänger et al., 2012, 2013; Szymanski et al., 2017b; Gvirts and Perlmutter, 2020; Müller et al., 2021a; Novembre and Iannetti, 2021; Reinero et al., 2021; Gugnowska et al., 2022). This becomes particularly clear when we consider, for example, the coupling between brains and sounds elicited by musical instruments (guitars), as illustrated in the work by Müller and Lindenberger (2019). Figure 1 shows the coupling between brains and musical instruments in a guitarist duet during free improvisation. To investigate phase coupling between EEG and auditory signals, the amplitude of the auditory signal was firstly averaged within the four frequency ranges: low (50–250 Hz), middle (250–500 Hz), high (500–2,000 Hz), and whole range (50–2,000 Hz). Thereafter, directed coupling measured by the Integrative Coupling Index (ICI) was determined between EEG and transformed auditory signals in four frequencies of interest (1.25, 2.5, 5, and 10 Hz; see Müller and Lindenberger, 2019, for more details). Figure 1A shows the ICI within (left panel) and between (right panel) the brains and instruments of the guitarists’ A and B at the delta frequency (1.25 Hz). To clarify, brain maps were created that are influenced by the signals from individual guitars and also display reciprocal effects of the two musicians’ brains on each other. Out- and in-strength were calculated as a sum of outgoing (from one node to all others) and incoming (to one node from all others) connections, respectively. Brain maps of out-strength (Figure 1B) and in-strength (Figure 1C) distribution within and between the brains as well as an in-strength distribution from the guitar to the brain (Figure 1D) and an out-strength distribution from the brain to the guitar (Figure 1E) are depicted. For this representation, the four guitar nodes were added together. It can be seen that connections from brain A to brain B go from temporoparietal regions in A to temporoparietal and above all right- and mid-frontal regions in B, while the connections from brain B to brain A go from right temporal regions in B to left temporoparietal regions in A. At the same time, the brain of guitarist A receives the connections from guitar A in frontotemporal regions (especially left) and at temporoparietal brain regions from guitar B, whereas the brain of guitarist B receives the connections from both guitars in temporoparietal and occipital brain regions. Thus, the guitars address brain regions in the two brains that are interconnected as well as those that are not. In addition, there are connections between brain regions that are not connected with the guitars. This could indicate that areas that are connected between brains are not affected by the guitars. It is evident that the two guitars have different effects on the two brains and that the reciprocal effects of the two brains on each other are different. This challenges the claim that synchronization between brains is simply a result of a common perceptual input and/or a common motor output. Recently, in pianist duets, while keeping sensory input and movements comparable across conditions as well as during musical pauses without sensory input or movement, it has been shown that inter-brain synchrony does not merely depend on shared sensorimotor impact but can also emerge endogenously, from aligned cognitive processes supporting social interaction (Gugnowska et al., 2022).

Interesting evidence found in an EEG hyperscanning study on analgesia is related to social touch, where romantic partners were assigned the roles of the target (pain receiver) and observer (pain observer). It has been found that hand-holding during pain administration increases the brain-to-brain coupling between the central regions of the pain receiver and the right hemisphere of the pain observer (Goldstein et al., 2018). This increase in brain-to-brain coupling cannot be explained through a common sensory input, because the pain was administered to only one participant. It was also found that brain-to-brain coupling correlated significantly with the target’s analgesia magnitude and the observer’s empathic accuracy (Goldstein et al., 2018). In other words, enhanced inter-brain synchrony resulted in two distinct subjective experiences, that is, analgesia in one case and empathy in the other. Recently, this evidence was also confirmed in hyperscanning animal studies using different approaches (Kingsbury et al., 2019; Omer et al., 2019; Zhang and Yartsev, 2019). In a mouse study with microendoscopic calcium imaging to record neural activity from thousands of neurons, two animals, showing high interbrain correlations between the dorsomedial prefrontal cortex (dmPFC) neurons during social vs. non-social behavior, significantly reduced interbrain synchrony when tested within the same physical environment (constant ambient noise and lighting) with a barrier abolishing social interaction (Kingsbury et al., 2019). In another study using extracellular electrophysiology recordings in two bats, high inter-brain synchrony measured by local field potential correlations between two bats persisted even when one of the bats was only observing the interaction of his companion with a third bat that shared their enclosure (Zhang and Yartsev, 2019). Interestingly, Kingsbury and Hong (2020) hypothesized that “…a subject’s attentional state may be compared with the estimated attentional state of an interacting partner by some circuit. By computing the synchronization of self and inferred attentional states across individuals, such a circuit could shape behavior based on the estimated synchrony of their attentional states. Although such a mechanism has not been tested, it is possible to determine whether any neural components encode the inter-brain synchronization of specific neural processes” (Kingsbury and Hong, 2020, p. 663). It is an interesting hypothesis, and perhaps such circuits do exist, but I would expect hyper-brain assemblies to be initially heterogeneous, distributed over the entire brain, and generally supportive of any kind of social behavior (cf. Prounis and Ophir, 2020). Nevertheless, further research in this direction is needed to properly understand the dynamics and origins of inter-brain synchronization and to effectively address the challenges of brain-to-brain interaction.

Interestingly, in a recent hyperscanning neurofeedback study, it has been shown that participants can learn to adjust their brain activity by using inter-brain synchrony as a neurofeedback feature (Müller et al., 2021b). Moreover, Basso et al. (2021) recently proposed the* synchronicity hypothesis of dance*, which states that humans dance to enhance both intra- and inter-brain synchrony. In other words, enhancement of intra- and inter-brain synchrony is supposed to be the desired result of a coordinated action, namely dancing. Thus, the authors hypothesized that dance evolved as a spontaneous process to drive coherent neural activity between brain regions within the brains of dancers and also between their brains. At the same time, dance is controlled or affected by the brain and thus emerges from the brain (Basso et al., 2021). This is in line with the aforementioned study on synchronization between musicians and instruments by Müller and Lindenberger (2019), where the authors stated that “…the instrument’s sound is a result of the musician’s behavior, which is based on sensorimotor synchronization and action. At the same time, this sound influences the behavior of musicians through auditory sensory pathways and is in this sense an actor. In our view, music improvisation and interaction can be understood only when considering both bidirectional influences” (Müller and Lindenberger, 2019, p. 9). As hypothesized by Novembre and Keller (2014), *action-perception coupling* when playing music in an ensemble facilitates the capacity to generate predictions of the respective musician’s own as well as others’ actions and to form representations of actions produced by others, and to integrate them with self-generated actions in real-time.

### The Inter- or Hyper-Brain Cell Assembly Hypothesis

From everyday life, we know that social activities, such as making music, dancing, acrobatics, etc., can and must be learned and practiced to be effective and smooth. During learning (by repeated social activity), unnecessary degrees of freedom of interpersonal interaction are eliminated, with positive effects on smooth movement and motor skills. As mentioned above, there is an intrinsic relation between oscillatory activity, neural cell assemblies, and behavioral or cognitive entities. This relation also concerns inter-brain oscillatory activity or synchrony. In this context, we suggest an *inter- or hyper-brain cell assembly hypothesis* that states that cell assemblies can be formed between brains as well, following roughly the same rules as within brains. Probably, I would rephrase the Hebbian rule in accordance with this context: “what wires together, fires together” (“wire” here in the sense of inter-brain connectivity or synchrony), indicating that cell assemblies that are interconnected between the brains by means of inter-brain synchrony ignite simultaneously or synchronously within these brains. This also means that cell assemblies within brains that form during an interaction and are synchronized with each other through between-brain interaction or synchrony will also gain precedence during repeated joint activity. This leads to the formation of the so-called inter-brain or rather *hyper-brain cell assemblies* (because both intra- and inter-brain connections are involved), which interconnect the two (or more) brains and lead to the joint firing of neuronal elements in these brains or in the common hyper-brain cell assembly. We also assume that such hyper-brain cell assemblies can be generalized with respect to other individuals or brains, albeit with some interpersonal variability.

**Figure 2:**
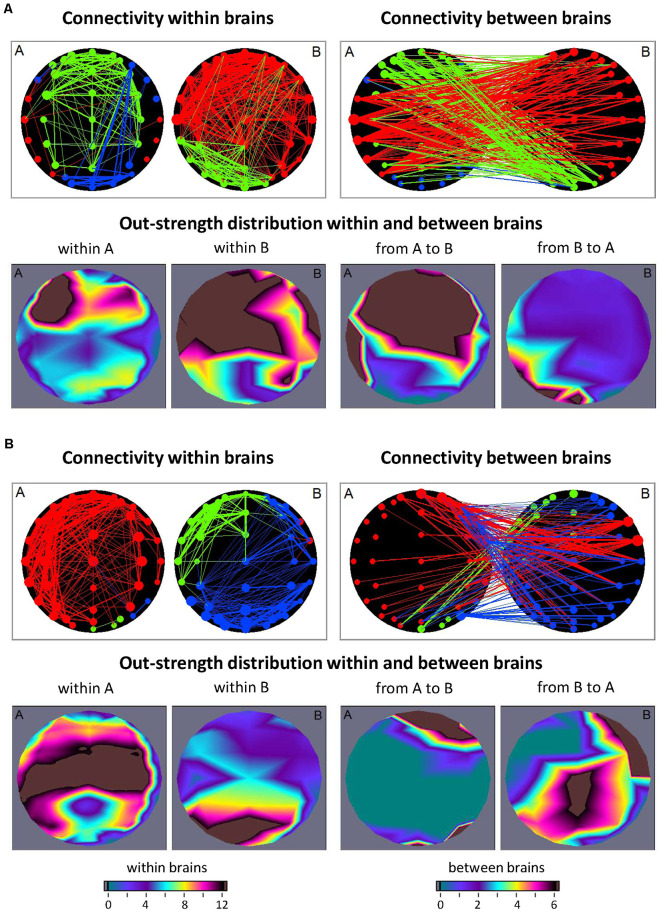
Connectivity within and between the brains and corresponding out-strength distributions during free guitar improvisation. **(A)** Connectivity maps and out-strength distributions in the first time window. In the first row, the left panel represents the connectivity within the brains, and the right panel represents the connectivity between the brains. The size of the circles represents the strength of the nodes (electrodes) and connectivity strength is coded by line thickness. The three different modules are coded by color. In the second row, the two maps on the left represent the topological distribution of the out-strengths within the brains of the two guitarists, and the right ones display the topological distribution of the out-strengths going from guitarist A’s brain to guitarist B’s brain and* vice versa*. **(B)** Connectivity maps and out-strength distributions in the second time window. Connectivity maps within and between the brains and corresponding out-strength distribution maps are represented in the same way as in **(A)**.

In the studies with guitarist duets (Sänger et al., 2012; Müller et al., 2013) and quartets (Müller et al., 2018b), so-called *hyper-brain modules* comprising nodes in two or more brains were found. Such hyper-brain modules or communities, in which the connections within the modules are the strongest, will be considered as one of the possible representations of the hypothesized hyper-brain cell assemblies. As an example, consider a guitarist duet during free improvisation. Figure 2 shows two different 2-s sequences during the improvisation. Analogous to the previous example (Figure 1) directional coupling (ICI) was calculated within and between the brains in the delta (1.25 Hz) frequency. The entire hyper-brain network was then examined using modularity analysis. The modules are labeled in different colors. In the first sequence (Figure 2A), the hyper-brain network consists of three modules, while one of them (marked in blue) consists of nodes within one brain (guitarist A), and the other two modules (red and green) are hyper-brain modules that are represented in both brains. But what is striking here is the different network structures in the two hyper-brain modules. If the electrodes or the nodes in the green module are strongly connected both within and between the brains, the connections in the red module within the brain of guitarist A (temporal nodes) are very weak, but these nodes send strong connections to the brain of guitarist B, which is itself strongly interconnected. In other words, the red module comes about through the strong connections between the brains and through the strong connections within the brain of guitarist B. In the second sequence (Figure 2B), there are also three modules, but this time all three are hyper-brain modules. Notably, all three modules show strong connections either within the brain of guitarist A or within the brain of guitarist B. The remaining nodes in these modules are weakly connected within the brains but respectively connected to a different brain as if these nodes play a subordinate role within the brains but a connector role between the brains. As indicated by the brain maps of out-strength distribution, the fronto-central and temporal regions play a leading or important role in both sequences. By definition, the connections between the nodes within the modules are the strongest, so the hyper-brain modules or communities comprising nodes located in different brains must have an important functional meaning (Müller et al., 2021a). These strong connections within the hyper-brain module(s) are important for information transfer between the brains (and also within them) and for simultaneous and probably synchronized firing ignition of neural cells within these brains. As shown, these hyper-brain modules can have different modularity structures, which change in time dependent on the situation or interaction circumstances. Hyper-brain modules are not equal to hyper-brain cell assemblies, but nevertheless, such hyper-brain modules may represent a prototype of cell assemblies that can play a central role in inter- or hyper-brain communication.

Similar ideas were recently conveyed in a hypothesis article by Shamay-Tsoory (2021). She refers to *interbrain plasticity* or *interaction-based learning*, where the term *interbrain plasticity* is used as a metaphor representing the capacity of the inter-brain networks (based also on intra-brain connectivity) to rearrange their functional structure in response to interaction-based learning. “Notably, if brain regions hold the capacity to coordinate their activity within a brain, it is possible that brain regions coordinate their activity *between brains*” (Shamay-Tsoory, 2021, p. 4). From a historical perspective, it is worth mentioning Varela’s ideas or concept of interbeing (Varela, 1999) as well as the related concept of “genuine intersubjectivity” or “extended consciousness” (Froese, 2018; Valencia and Froese, 2020), which proposed inter-brain phase synchronization of neural oscillations as a candidate mechanism for the conscious extended mind, linking the idea of large-scale integration within brains to inter-brain synchrony.

In the aforementioned hyperscanning mouse study, Kingsbury et al. (2019) showed that interbrain activity correlations during competitive interaction arise from single-cell dynamics and that cells in dominant and subordinate mice encode subject and opponent information differently, whereby dominant mice exert a greater influence on interbrain synchrony than subordinates do. Moreover, neuronal cells in dominant animals respond more to subject behaviors compared to cells in subordinates, while cells in subordinates respond more to opponent behaviors compared to cells in dominants. The authors concluded that interbrain synchrony depends specifically on subsets of neurons that separately encode behaviors of the subject animal and those of the interacting partner (i.e., social behaviors of self and others) and allow each brain to represent a common repertoire of the behavior of both interacting animals (Kingsbury et al., 2019). Although the measurement method used in this study (i.e., microendoscopic calcium imaging) does not allow conclusions about neuronal firing to be drawn, the results of the study speak about correlated activity of cell assemblies in two brains of the test animals in social interaction. In this regard, the study by Zhang and Yartsev (2019), in which electrophysiological local field potentials were measured in bats, is more informative with respect to neuronal firing. They observed that the 30–150 Hz local field potential was linked to aggregate local spiking activity and that the degree of interbrain neural correlation covaried with the extent of social interactions (Zhang and Yartsev, 2019). Both these studies indicate correlated neural activity on the cell level. It remains to be seen whether cell assemblies that are interconnected between brains *via* inter-brain synchrony or correlated neural activity ignite simultaneously or synchronously within these brains. The suggested hyper-brain cell assembly hypothesis predicts or assumes a simultaneous firing of neural cells in two (or more) brains.

From a synergetic point of view, a hyper-brain assembly represents a system or superordinate system that is controlled at the macro level by certain order parameter(s). The order parameter determines the behavior of the neuronal elements in a hyper-brain assembly and thus “enslaves” the behavior of the individual parts. At the same time, the individual parts on the micro-level of neuronal elements (neurons or synapses) can influence the structure and dynamics of hyper-brain assembly as a whole in accordance with the *circular causation* rule (Haken, 2006, 2016). Importantly, this principle of circular causation works not only within individual brains but also in a hyper-brain network or assembly binding two or more brains together that function as a superordinate system or superorganism (cf. Müller et al., 2021a).

## Within- and Cross-Frequency Coupling and Related Hyper-Brain Assembly

**Figure 3:**
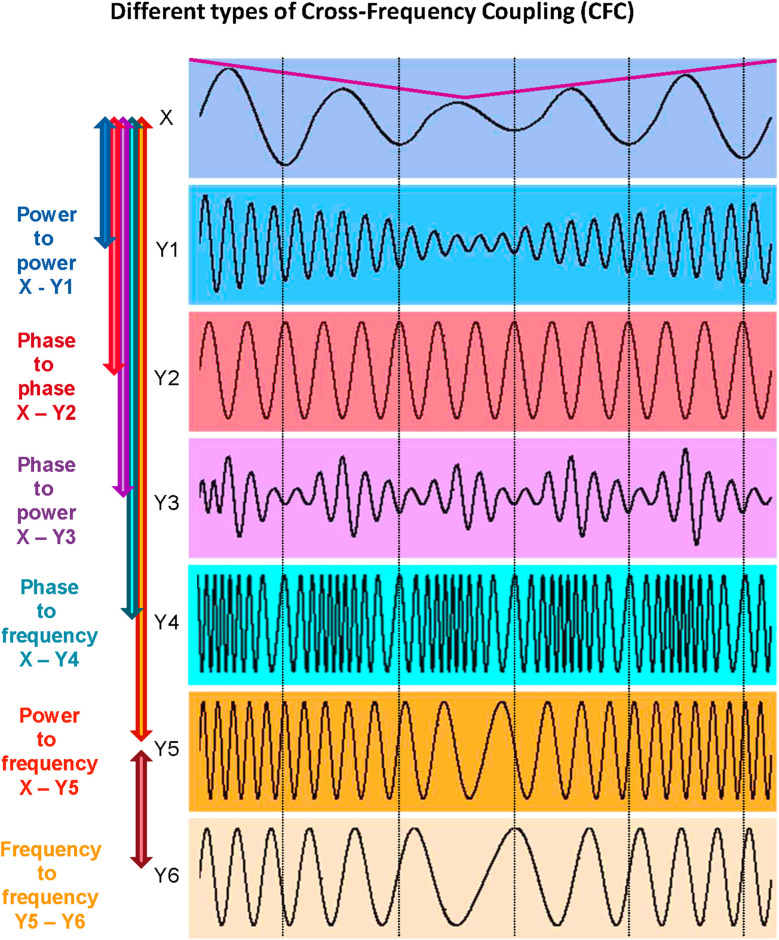
Different types of cross-frequency coupling (CFC). *Power-to-power CFC* can be identified between signals *X* and *Y1*: Signal *Y1* about five times higher frequency than that of signal *X* shows slow amplitude modulations over time like signal *X* (indicated by the purple line). *Phase-to-phase CFC* can be identified between signals *X* and *Y2*: Signal *Y2* shows 3:1 phase-to-phase coupling with signal *X*, i.e., one oscillation period of signal *X* corresponds to three periods of signal *Y2*. *Phase-to-power CFC* can be identified between signals *X* and *Y3*: Signal *Y3* shows fast amplitude modulations that are related (or coupled) to the phase of signal *X*. *Phase-to-frequency CFC* can be identified between signals *X* and *Y4*: Signal *Y4* shows frequency modulations that are coupled with phase changes of signal *X*. *Power-to-frequency CFC* can be identified between signals *X* and *Y5*: Signal *Y5* shows frequency modulations that are related to, or coupled with, the slow amplitude modulations of signal *X* (purple line). *Frequency-to-frequency CFC* can be identified between signals *Y5* and *Y6*: Signal *Y6* shows slower but similar frequency modulations as the signal *Y5*. The different types of CFC are not mutually exclusive (cf. Jensen and Colgin, 2007). For instance, slow amplitude modulations of signal *X* are coupled not only with the amplitude changes of signal *Y1* but also with frequency changes of signals *Y5* and *Y6*, which are coupled in their frequency modulations at the same time (adapted from Jirsa and Müller, 2013).

It has already been shown that synchronization by oscillation and related binding phenomena play a crucial role in neural communication. Neural interaction can occur at the same or at different frequencies and can be indexed by within- and cross-frequency coupling (WFC and CFC, respectively). It has been suggested that WFC and CFC can represent information flows within and between neural cell assemblies, respectively (Buzsáki and Draguhn, 2004; Buzsáki, 2006; Müller et al., 2016, 2019b). The interaction between different characteristics of the signals indicated by different WFC and CFC measures adds another dimension to understanding complex neural dynamics and neuronal networks (Jirsa and Müller, 2013). As reported by Buzsáki and Draguhn (2004), neuronal cell assemblies oscillating synchronously at different frequencies provide an efficient basis for integrative processes in the brain. CFC, allowing accurate timing between different oscillatory rhythms, maybe one of the mechanisms underlying the re-integration of these separated information flows and allowing for communication between different cell assemblies (Canolty et al., 2006; Klimesch et al., 2008; Doesburg et al., 2009; Canolty and Knight, 2010). As suggested by Buzsáki, “…frequency locking can occur between any two or more oscillators with an integer period relationship. In principle, virtually infinite numbers of combinations are possible but the limited number of classes of oscillators that can be simultaneously present in the same neuronal substrate puts severe constraints on the possible numbers of combinations” (Buzsáki, 2006, p. 354). It should be added here that besides the aforementioned *phase-to-phase* CFC with an integer period relationship, there are other CFC forms that can play an important role in neuronal and other biological or social interactions (cf. Jensen and Colgin, 2007; Jirsa and Müller, 2013; Hyafil et al., 2015), such as: (i) *power-to-power*; (ii) *phase-to-power*; (iii) *phase-to-frequency*; (iv) *envelope- (*or* amplitude-) to-frequency*; and (v) *frequency-to-frequency* CFC. These CFC types are schematically presented in Figure 3 (cf. Jirsa and Müller, 2013).

There is increasing evidence that the phase-amplitude modulation occurs most often—it has been found both in animals and humans in the entorhinal and prefrontal cortices, in the hippocampus, and in distributed cortical areas (Mormann et al., 2005; Canolty et al., 2006; Tort et al., 2008, 2009, 2010; Cohen, 2008; Osipova et al., 2008; Cohen et al., 2009a, b; Colgin et al., 2009; Axmacher et al., 2010a, b; Voytek et al., 2010; Munia and Aviyente, 2019). In particular, it was observed that the phase of low-frequency oscillation (e.g., theta) modulates power in high-frequency oscillations (e.g., gamma), with stronger modulation mostly occurring at higher theta amplitudes (Canolty et al., 2006; Jensen and Colgin, 2007; Cohen, 2008; Tort et al., 2008, 2009; Doesburg et al., 2009; Canolty and Knight, 2010; Kendrick et al., 2011; Colgin, 2015; Amemiya and Redish, 2018). Besides the CFC between low-frequency phase and high-frequency amplitude (e.g., theta-gamma), there is evidence that phase-amplitude CFC also exists between the low-frequency bands (e.g., delta-theta, delta-alpha, and theta-alpha; see Witte et al., 2000; Lakatos et al., 2005; Schack et al., 2005; Cohen, 2008; Isler et al., 2008). Furthermore, it was reported that the high-frequency gamma amplitude can also be modulated by the alpha phase (Jensen and Colgin, 2007; Cohen et al., 2009a; Voytek et al., 2010). Lakatos et al. (2005) found that the delta (1–4 Hz) phase modulates theta (4–10 Hz) amplitude, and the theta phase modulates gamma (30–50 Hz) amplitude in the primary auditory cortex of awake macaque monkeys. Based on their findings, the authors introduced a hypothesis on the “hierarchical” organization of EEG oscillations suggesting that the amplitude of the oscillations at a characteristic frequency is modulated by the oscillatory phase at the lower frequency (Lakatos et al., 2005). Furthermore, as mentioned above, there is also clear evidence that not only the amplitude of the high-frequency oscillations is modulated by the oscillatory phase at a lower frequency but also that phases of both these oscillations can be related to each other (e.g., theta-gamma phase-to-phase coupling; see Schack et al., 2002; Schack and Weiss, 2005). Moreover, other types of CFC (e.g., amplitude-to-amplitude or envelope-to-envelope, frequency-to-frequency, and amplitude/envelope-to-frequency) may occur and have indeed been observed (De Lange et al., 2008; Witte et al., 2008, 2011; Jirsa and Müller, 2013; Hyafil et al., 2015). All these interaction patterns exist simultaneously in biological signals and thus provide a more complete picture about information processing in the brain or other biological systems and subsystems (Jirsa and Müller, 2013). It is worth noting here that all these CFC forms are associated with, or implicated in, the relative coordination described above with respect to the important work by Erich von Holst many decades ago (cf. Müller et al., 2011).

**Figure 4:**
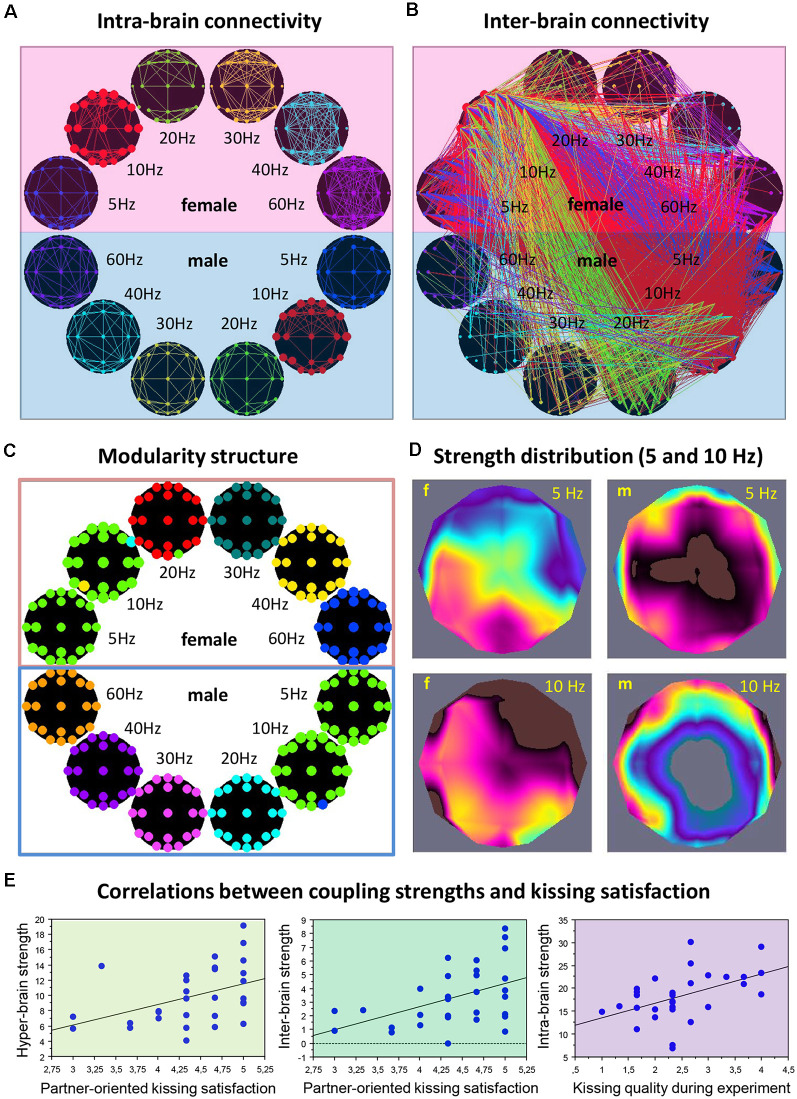
Representation of the hyper-brain network and the theta-alpha subnetwork during kissing. **(A)** Intra-brain connectivity. The brain maps represent connectivity at six different frequencies within the female and male partners’ brains, respectively. **(B)** Intra-brain connectivity. The brain maps represent the between-brain connectivity within and between the six frequencies. It can be seen that the strongest connections are found in the theta (blue) and alpha (red) frequency bands, and between them. **(C)** Modularity structure. Modularity analysis revealed nine different modules. The biggest module is the so-called theta-alpha module or subnetwork comprising 5- and 10-Hz nodes in both female and male brains. Other modules represent different frequencies in both brains separately. **(D)** Out-strength distribution of theta (5 Hz) and alpha (10 Hz) nodes within the female and male brains. The topological distribution of coupling strength represents the overall within- and cross-frequency connectivity within the theta-alpha subnetwork for female and male brains, respectively. **(E)** Correlations between coupling strengths and kissing satisfaction and kissing quality. Partner-oriented kissing satisfaction correlated significantly positively with the hyper- and especially inter-brain strengths determined for 5-Hz oscillation nodes. Kissing quality during the experiment correlated significantly positively with intra-brain strength determined for 10-Hz oscillation nodes (adapted from Müller and Lindenberger, 2014 and Müller et al., 2021a).

**Figure 5:**
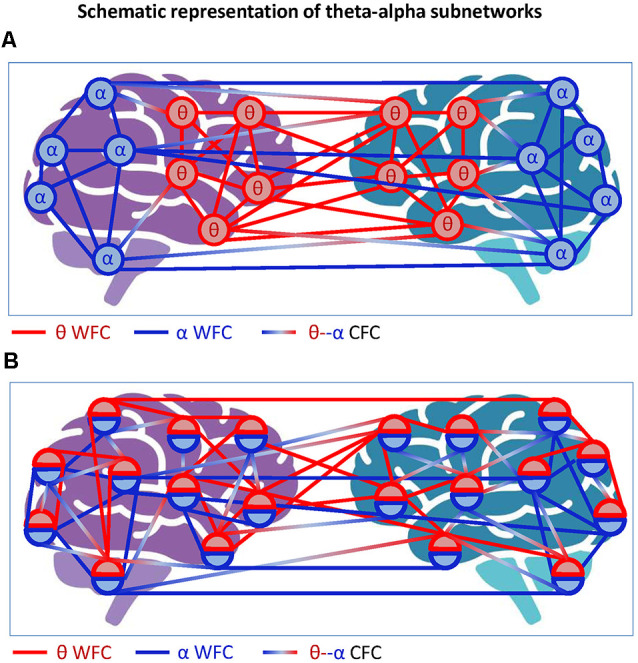
Schematic representation of theta-alpha subnetworks. **(A)** Theta-alpha subnetwork as two hyper-brain modules oscillating at different frequencies. The two hyper-brain modules oscillating at different frequencies (θ and α) are coded by colors (blue and red). The coupling within the modules is given by WFC and the coupling between the modules by CFC. **(B)** Theta-alpha subnetwork with nodes oscillating at two different frequencies each. The two hyper-brain modules oscillating at different frequencies (θ and α), also coded in blue and red, are distributed across two brains, whereby each node belongs to both modules. The coupling within the modules is given by WFC and the coupling between the modules by CFC.

It is also justified to assume that hyper-brain cell assemblies can make use of CFC. An example of a hyper-brain cell assembly based on WFC and CFC could be the so-called theta-alpha networks detected when couples kissed (Müller and Lindenberger, 2014). This is displayed in Figure 4. It can be seen that 5- and 10-Hz oscillation nodes are strongly interconnected within (Figure 4A) and especially between (Figure 4B) the partners’ brains. Modularity analysis revealed a hyper-brain module comprising these oscillation nodes in both brains (indicated in green in Figure 4C), which also shows different strength distributions in the two brains (see Figure 4D for details). Interestingly, hyper- and especially inter-brain strength determined for 5-Hz oscillation nodes (Figure 4E, left and mid panel, respectively) correlated significantly positively with partner-oriented kissing satisfaction, and intra-brain strength determined for 10-Hz oscillation nodes correlated significantly positively with self-reported kissing quality (Figure 4E, right panel). In other words, the main parts of the theta-alpha subnetwork (i.e., theta and alpha oscillatory nodes) have certain relations to the subjective feelings of the kissing subjects, that is, the hyper-brain module or cell assembly and its parts (intra- and inter-brain connection strengths) are related to social behavior outcomes. This hyper-brain assembly or theta-alpha subnetwork is based on phase-to-phase WFC and CFC, comprising connections both within and between the brains. The question that arises here: Can a subnetwork (i.e., the theta-alpha subnetwork) identified using modularity analysis be equated with a cell assembly? If we assume that neural cells within cell assemblies communicate with each other at the same frequency and that the CFC is responsible for the communication between the cell assemblies, then it can be supposed that the theta-alpha subnetwork contains (at least) two hyper-brain cell assemblies oscillating at theta and alpha frequencies that are strongly interconnected and form hierarchically organized structure or the theta-alpha subnetwork. Such a situation is schematically represented in Figure 5A.

Another possible scenario is depicted in Figure 5B, where each electrode or node contains two oscillations. In this case, we can speak of a common theta-alpha subnetwork comprising all nodes with their WFC and CFC links. This scenario, which probably corresponds more to the situation outlined in Figure 4, will be discussed in the next section with respect to multilayer networks, which are more suitable for such situations. At this point, we only note that if we consider the concept of hierarchical modularity or modularity at multiple topological scales (cf. Meunier et al., 2009, 2010), the scenarios shown in Figure 5 or mixed scenarios would or could occur.

**Figure 6:**
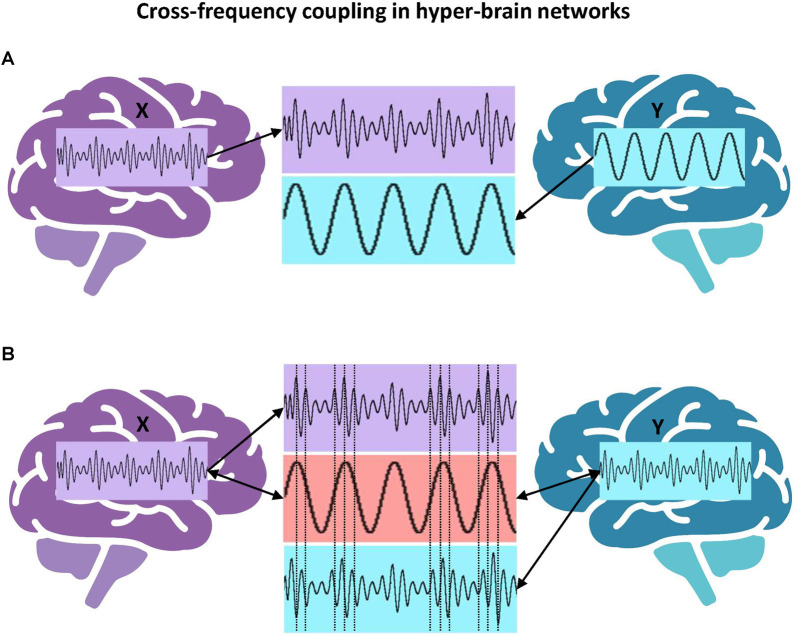
Cross-frequency coupling in hyper-brain networks. **(A)** Hyper-brain theta-gamma phase-amplitude CFC, scenario 1. The amplitude or envelope of signal X in the brain on the left is coupled to the phase of signal Y in the brain on the right. **(B)** Hyper-brain theta-gamma phase-amplitude CFC, scenario 2. Signals X and Y (in the brains on the left and right, respectively) are gamma oscillations, which are mostly out of phase (indicated by the vertical dotted lines in the middle) but their amplitude or envelope is modulated by the common theta rhythm, which can be either endogenous or exogenous.

Next, an example of phase-amplitude cross-frequency coupling with respect to the hyper-brain cell assembly hypothesis should be discussed. Figure 6A represents a scenario in which the amplitude of signal X in the brain on the left is coupled to the phase of signal Y in the brain on the right. If we follow the literature (Lisman, 2005; Buzsáki and Wang, 2012) and assume that the period of gamma activity is closely related to the firing activity of neurons, then one could speculate that the activity of spiking cells in the left brain occurs during certain periods of the theta phase in the right brain. Figure 6B represents another scenario of phase-amplitude cross-frequency coupling between two brains. In this case, signals X and Y (in the brains on the left and right, respectively) are gamma oscillations, which are mostly out of phase (indicated by the vertical dotted lines in the middle) but their amplitude or envelope is modulated by the common theta rhythm (cf. Buzsáki and Wang, 2012). Presumably, this theta rhythm could be located in the brain or brains (e.g., in the hippocampus) and accordingly induce the gamma oscillation cycles in two brains, or it could be purely virtual or originate from the environment. Importantly, in this case, the hyper-brain cell assembly activity represented by high-frequency gamma oscillation cycles is synchronized *via* the modulating activity of low-frequency theta oscillation. Recently, in a hyperscanning EEG study on pianists jointly performing duets, inter-brain synchrony was calculated by extracting the amplitude envelopes in five frequency bands (delta: 1–3 Hz, theta: 4–7 Hz, alpha: 8–12 Hz, beta: 13–30 Hz, and gamma: 30–40 Hz), band-pass filtering these envelopes in the frequency range of pianists’ planned and actually performed musical tempi (1–3 Hz), and finally by extracting the phase of these envelopes to calculate the aforementioned inter-brain synchrony indices. Practically, this approach corresponds to the scenario shown in Figure 6B, with the difference that the delta-gamma (instead of theta-gamma) phase-amplitude CFC was calculated, although the authors do not speak of CFC in their work (Gugnowska et al., 2022). Please note that gamma (and also other frequency) oscillations were modulated by the delta rhythm (the pianists’ planned and performed musical tempi), which can be induced either endogenously or exogenously.

In general, the combination of WFC and CFC has a stronger explanatory power because cortical, and more generally, biological systems never operate at only one frequency. Different modulations that take place continually in complex systems are interconnected and influence each other in order to adapt and sufficiently react to the constantly changing environment. An important question that exists and needs to be answered in this context is the question of the relationship between oscillatory and firing or spiking neuronal activity. However, as discussed earlier and indicated by Buzsáki (2010, p. 366): “Oscillators are also natural parsing and chunking mechanisms of neuronal activity because they have well-defined onsets and offsets with characteristic maximum and minimum spiking activity of the information-transmitting principal cells.”

## Hyper-Frequency and Multilayer Networks

**Figure 7:**
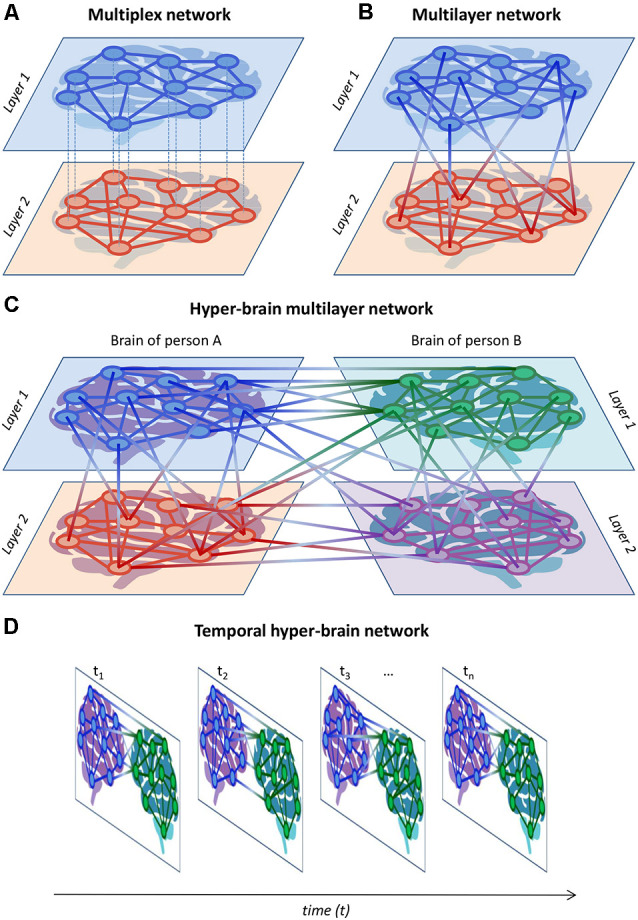
Schematic representation of multiplex, multilayer, and temporal networks. **(A)** Multiplex network. A two-layer single-brain multiplex network is represented. The layers can represent, for example, different frequencies or other brain attributes (e.g., structural and functional connectivity). **(B)** Multilayer network. A two-layer single-brain multilayer network is represented, where the nodes are connected within and between the layers. The connections within the layers can be given by WFC and those between the layers by CFC. **(C)** Hyper-brain multilayer network. A two-layer hyper-brain multilayer network is represented, where the nodes are connected within and between the layers as well as within and between the brains. In the case of an HFN, connections within the layers are given by WFC, connections between the layers are given by CFC, and connections between the brains are given both by WFC and CFC. The two layers of persons A and B can be considered as two different *aspects*. **(D)** Temporal hyper-brain network. Network structures can change across time and can be considered multiplex (if the temporal layers do not have any causal relationships or are not connected) or multilayer (if the temporal layers are causally dependent or connected) networks. For simplicity, the connections between the temporal layers were omitted but they would appear if the network was multilayer.

Networks including both types of couplings, WFC and CFC, were called hyper-frequency networks (HFNs) and were found within (Müller et al., 2016, 2019b) and between brains (Müller and Lindenberger, 2014) as well as in complex networks emerging during choir singing (Müller et al., 2018a, 2019a). Complex networks (e.g., HFNs) can be described as multiplex or multilayer networks that have a specific multidimensional or multilayer network organization (De Domenico et al., 2013, 2015, 2016; Boccaletti et al., 2014; Kivelä et al., 2014; De Domenico, 2017; Pilosof et al., 2017; de Arruda et al., 2018). Basically, multiplex networks can be considered as a special case of multilayer networks. In multiplex networks, the set of nodes in each layer is usually the same (but not always), and the nodes are connected within the layer but not between layers (e.g., in conventional functional connectivity analyses, the WFC at different frequencies can represent different layers that are not connected to each other). Multilayer networks are characterized by the existence of connections not only within the layers but also between them (e.g., by means of CFC). In the literature, there is no unified conceptualization of multiplex and multilayer networks. I present my view on these phenomena here and am mainly oriented towards the applicability of these concepts with regard to neuroimaging and hyperscanning research. In this context, HFNs can be described as multilayer networks, where WFC represents communication within different layers and CFC depicts communication between the layers (Brookes et al., 2016; Tewarie et al., 2016, 2021; De Domenico, 2017; Buldú and Porter, 2018; O’Neill et al., 2018; Tenney et al., 2021). Figures 7A,B exemplarily show complex two-layer single-brain networks in the form of multiplex (Figure 7A) and multilayer (Figure 7B) networks. It can be seen that in multilayer networks as compared to multiplex networks, there are connections between the layers that can be accomplished by CFC. In the case of hyper-brain multilayer networks, there are, in addition, connections between the brains that can occur both within and between frequencies (see Figure 7C for details). The two layers of persons A and B and the two layers within their brains, representing WFC at different frequencies, can be regarded as two different *aspects* or *features* of the multilayer network (cf. Kivelä et al., 2014). To avoid confusion, the four layers in Figure 7C are also called *elementary layers*, indicating the affiliation to different aspects or features of the multilayer network (Kivelä et al., 2014). Furthermore, networks changing in time can also be considered as multiplex (if the temporal layers do not have any causal relationships or are not connected) or multilayer (if the temporal layers are causally dependent or interconnected) networks, as represented in Figure 7D (for simplicity, I have omitted the connections between the layers here so that the representation can be either multiplex or multilayer). These temporal or time-varying networks, whose edges have intrinsic dynamics with given characteristic time scales, represent the temporal evolution of a system or information spreading across time (Holme and Saramäki, 2012; Kivelä et al., 2014; Starnini et al., 2017; O’Neill et al., 2018).

There is a body of work in neuroscience on *functional connectivity dynamics* (FCD) or *network topology dynamics* (NTD) that uses temporal or time-varying connectivity networks to study changes in network structure and resulting network topology over time (Betzel et al., 2012, 2016; Chu et al., 2012; Calhoun et al., 2014; Hansen et al., 2015; Shen et al., 2015; Deco and Kringelbach, 2016; Müller et al., 2016, 2019b). This kind of connectivity network dynamics is also called *chronnectome*, focusing on identifying time-varying, but reoccurring, patterns of coupling among brain regions (Calhoun et al., 2014). It has been shown that the network structure and FCD or NTD are non-stationary and reveals a rich dynamic pattern, characterized by rapid transitions switching between a few discrete functional connectivity states (Betzel et al., 2012, 2016; Hansen et al., 2015; Shen et al., 2015; Müller et al., 2016, 2019b). Furthermore, analysis of the temporal fluctuations of HFN structure has revealed specific NTD, i.e., temporal changes of different GTA measures such as strength, the clustering coefficient (CC), characteristic path length (CPL), and local and global efficiency determined for HFNs in different time windows (Müller et al., 2016, 2019b). Moreover, it has been found that variability of these NTD metrics, measured by the standard deviation across time, correlated positively with perceptual speed scores, indicating that a more variable NTD increases performance in cognitive or at least perceptual-speed functioning and enhances adaptive capabilities of the system or individual (Müller et al., 2019b). A study comparing topological stability and graph characteristics of networks across time (ranging from 1 s to multiple hours) showed that functional networks were highly variable in the order of seconds and that stable network structures emerge after as little as 100 s duration. These conserved network structures or subnetworks were found to persist across different states and frequency bands, and the most common edges were markedly consistent, constituting a persistent network “core” (Chu et al., 2012). Networks changing in time can also be considered as assembly sequences; as hypothesized by Buzsáki (2010, p. 363): “…analogous to words and sentences in language, neuronal assemblies are organized by syntactical rules that define their first-order and higher-order relationships.” Thus, neuronal cell assemblies not only have a hierarchical or multidimensional structure representing different levels of organization but also the temporal development of these assemblies itself is hierarchically organized by specific rules and regularities.

**Figure 8:**
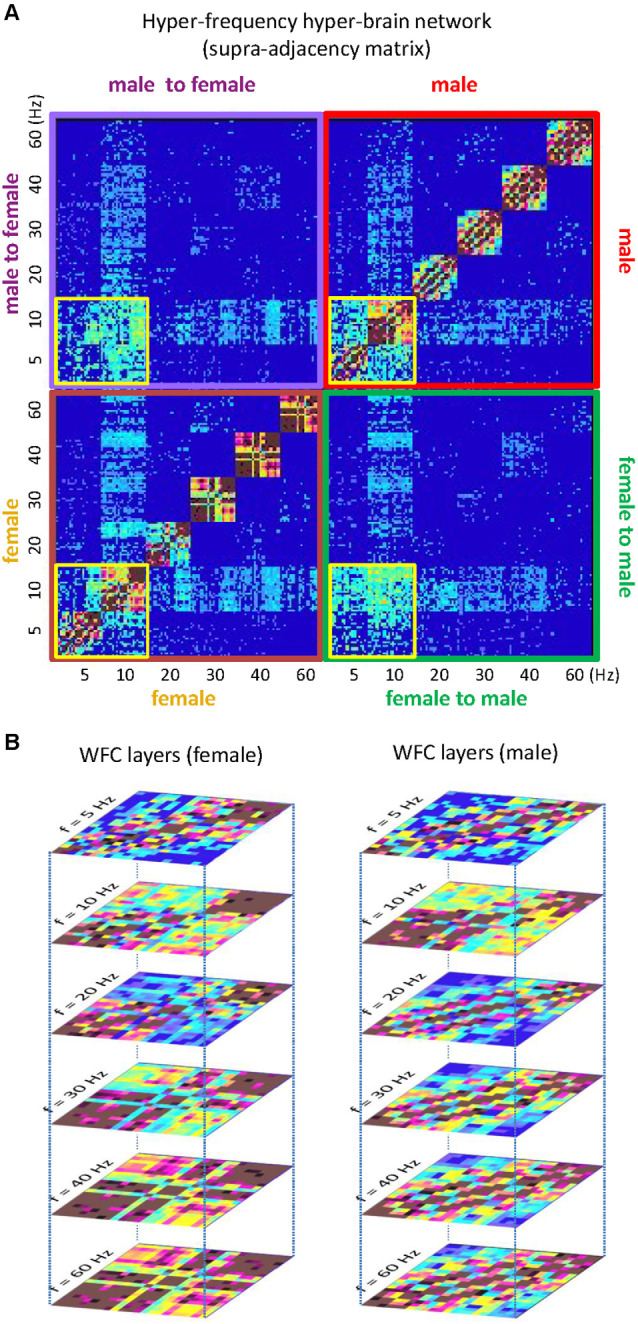
Hyper-frequency hyper-brain network during kissing. **(A)** Supra-adjacency matrix of the common network. The supra-adjacency matrix (252 × 252) comprises all connections between 21 electrodes and six frequencies (5, 10, 20, 30, 40, and 60 Hz) within (brown and red quadrats) and between (green and purple quadrats) female and male brains, respectively. WFC connections between all electrodes within the female and male brains are distributed along the main diagonal of the matrix. The electrodes for each frequency are organized in anterior-posterior order from left to right: Fp1, Fpz, Fp2, …,O1, Oz, and O2. WFC connections between the brains are distributed along diagonals within the green and purple quadrats. The 5- and 10-Hz nodes (indicated by four smaller yellow squares) represent the so-called theta-alpha subnetwork. The alpha frequency (10 Hz) has the most connections and serves a cleaving or pacemaker function in the common network. **(B)** WFC layers of the female and male subnetworks or aspects. The WFC subnetworks are presented here in the form of a six-layer structure that is depicted in A along the diagonal. The connections between the layers within the female and male brains are represented by remaining edges in brown or red quadrats in A. The two other quadrats in A (green and purple) represent connections (WFC and CFC) between the brains.

In a number of studies, it has been shown that multilayer networks and HFNs can also be represented as supra-adjacency matrices, where conventional GTA tools can be used to investigate the network properties (Kivelä et al., 2014; Müller and Lindenberger, 2014; Brookes et al., 2016; De Domenico et al., 2016; Müller et al., 2016, 2019b; De Domenico, 2017). Figure 8 illustrates a supra-adjacency matrix of a hyper-frequency hyper-brain network emerging during kissing. Practically, it is another representation of the network depicted in Figure 4 above. This matrix originally included 254 nodes (cf. Müller and Lindenberger, 2014) but, for simplicity, we removed two nodes indicating the two EMG lip responses of the female and male partners and reconstructed the matrix only on the basis of EEG signals oscillating at six different frequencies (5, 10, 20, 30, 40, and 60 Hz). Thus, the reconstructed supra-adjacency matrix (252 × 252) comprises all connections between 21 electrodes (organized in anterior-posterior order from left to right: Fp1, Fpz, Fp2,…, O1, Oz, and O2) and six frequencies within (brown and red quadrats) and between (green and purple quadrats) female and male brains, respectively (see Figure 8A). WFC connections between all electrodes within the female and male brains are distributed along the main diagonal of the matrix. WFC connections between the brains are distributed along diagonals within the green and purple quadrats. The 5- and 10-Hz nodes (indicated by the four yellow smaller squares) represent the so-called theta-alpha subnetwork (described above in Figure 4) comprising both WFC and CFC connections within and between brains, respectively. Moreover, the alpha-frequency (10 Hz) nodes have the most connections and serve a cleaving or pacemaker function in the common hyper-brain HFN. The six WFC subnetworks within the female and male brains represent the six layers of corresponding brains (see Figure 8B). The connections between the layers within the female and male brains are represented by the remaining edges in the brown or red quadrats in Figure 8A. The two other quadrants in Figure 8A (green and purple) comprise all connections (WFC and CFC) between the brains. As mentioned above, the multilayer network represented or organized in the form of a supra-adjacency matrix can be analyzed using the conventional GTA tools but also using other tools based on tensor algebra or tensor decomposition methods (Boccaletti et al., 2014; Kivelä et al., 2014; De Domenico et al., 2016; Cozzo et al., 2018; de Arruda et al., 2018).

In a choir study, it has been shown that physiological systems (respiratory, cardiac, and vocalizing) while singing are differently supported by WFC and CFC, whereby CFC connections are particularly strong when the choir sings a canon in parts, apparently supporting the interaction and coordination of the different canon entries. The balance between WFC and CFC provides effective HFN or multilayer network topology, allowing the choir to function as a superordinate system or superorganism (Müller et al., 2018a, 2019a). It has also been reported that HFNs, and thus multilayer networks, possess small-world network topology and exhibit different network topology dynamics, which can vary as a function of age, disease, or cognitive performance (Brookes et al., 2016; Müller et al., 2016, 2019b; Tewarie et al., 2016, 2021; De Domenico, 2017; O’Neill et al., 2018; Tenney et al., 2021) or with respect to different conditions of interpersonal action coordination (Müller and Lindenberger, 2014; Müller et al., 2018a, 2019a).

## Network Physiology and Neural Network Dynamics

As noted by Buzsáki (2010, p. 362), “With Hebb’s cell assembly hypothesis, it appeared that cognitive neuroscience had established a comprehensive research program to link psychological and physiological processes.” The biological system is an integrated network with different types of interaction between the elements or subsystems operating as a whole. These subsystems continuously interact through various feedback loops and across different spatio-temporal scales to optimize and coordinate their function (Bartsch et al., 2015; Ivanov, 2021). Physiological systems and organisms exhibit complex dynamics that transiently change over time under different physiological states (Faes et al., 2014; Liu et al., 2015; Ivanov et al., 2016; Rizzo et al., 2020; Ivanov, 2021). These physiological states are characterized by specific network structures and coupling strengths between systems and subsystems, demonstrating a robust interplay between network topology and function (Bashan et al., 2012). Plamen Ivanov and colleagues introduced the concept of time delay stability (TDS) to identify and quantify network connectivity among physiological systems and proposed a system-wide integrative approach that may facilitate the development of a new field, Network Physiology (Bashan et al., 2012; Ivanov et al., 2016; Ivanov, 2021). In one study, they investigated dynamical network interactions between six physiological systems represented by 10 network nodes: cardiac and respiratory activity, chin muscle tone, leg and eye movements, and spectral brain activity in the five frequency bands: δ (0.5–3.5 Hz), θ (4–7.5 Hz), α (8–11.5 Hz), σ (12–15.5 Hz), and β (16–19.5 Hz) (Bashan et al., 2012). The authors found that transitions between physiological states were associated with changes in network topology: during deep sleep (DS), the network consisted mainly of brain-brain links, while with transition to light sleep (LS), links between other physiological systems emerged and the network became highly connected. In another study using the TDS measure, the coupling between cortical rhythms and peripheral muscle (chin and leg muscle tone) activation rhythms was investigated during sleep and awake states (Rizzo et al., 2020). They showed that cortico-muscular coupling was stronger during wake, weaker during rapid eye movement (REM) sleep and LS, and weakest during DS. Furthermore, they found that cortical rhythms (EEG) preferentially coupled with specific muscle rhythms (measured *via* electromyography, EMG) predominantly at the same frequency (i.e., WFC): (i) γ1 (20–33.5 Hz) and γ2 (34–98.5 Hz) brain and muscle rhythms strongly communicated with each other, particularly during wake and REM sleep; and (ii) slower rhythms (δ, θ, α, σ, and β) became strongly involved in the interaction between brain and muscles (both chin and leg) during REM sleep, LS, and DS. A pronounced transition from low brain network connectivity in DS and REM to high connectivity in LS and wake was also observed in a study by Liu et al. (2015). They also reported that different brain areas exhibited different network dynamics to achieve differentiation in function during different sleep stages, which was also frequency-specific (Liu et al., 2015). In another study using entropy-based measures to investigate the network dynamics of the heart and different spectral brain sub-systems (varying in the five frequency bands: δ, θ, α, σ, and β), it has been shown that the β EEG power node, acting as a hub in the brain–heart network, sent the largest part of the information coming from the brain–brain subnetworks to the heart and, at the same time, forwarded the information arriving from the heart system to the other brain subsystems (Faes et al., 2014).

In the aforementioned hyperscanning kissing study (Müller and Lindenberger, 2014), the coupling between the lip EMG and EEG brain waves was investigated within and between the individuals using WFC and CFC measures. The lip-brain coupling proved to be highest at the same frequency (i.e., 60 Hz) both within and between the individuals. Furthermore, the 10-Hz EEG oscillations, serving a cleaving or pacemaker function in the common hyper-brain network of the kissing couples, showed high CFC with the lip EMG of both participants (Müller and Lindenberger, 2014). However, the lip EMG nodes did not show any significant correlations with partner-oriented kissing satisfaction or self-reported kissing quality. Presumably, the theta-alpha subnetworks discussed above play a more prominent role in subjective feelings when kissing. Nevertheless, it is important to know how the cell assemblies or hyper-brain cell assemblies are related to other systems or subsystems. Due to the fact that different systems or subsystems in an organism (respiration, cardiac, etc.) function in a different (mostly lower) frequency range than the brain, the cross-frequency coupling may play an important role here.

In the choir study, with 11 singers and a conductor, directed coupling measures showed strong, mostly unidirectional influences of the conductor on the choir members, indicating that changes in the oscillatory activity of respiration and heart rate variability occurred in the conductor before the choir members, in accordance with the conductor’s functional role. Furthermore, the choir members singing different parts of a song or canon could be partitioned into different modules or communities (in accordance with the parts sung, but not when singing these in unison; Müller and Lindenberger, 2011; Müller et al., 2018a). Thus, coupling strengths between systems and subsystems among the individuals involved in a coordinated activity (i.e., singing in a choir) also demonstrated a robust interplay between network topology and function (cf. Bashan et al., 2012). How such physiological systems or subsystems are linked to hyper-brain cell assemblies and how they contribute together to coordinated behavior remains to be seen.

## Future Research: Proving The Hyper-Brain Cell Assembly Hypothesis on Different Levels

As shown, the hyper-brain networks based on intra- and inter-brain synchronization or coupling often have a complex and multilayer organization. This network organization is based on WFC and CFC, which also connect different systems and subsystems when two or more subjects interact or communicate with each other. It is proposed here that hyper-brain cell assemblies are capable of playing an important role in controlling the neural processes that take place in multibrain or multisystem interaction.

How can the proposed hyper-brain cell assembly hypothesis be tested? There are certainly different levels to test. The first level that comes to mind is the cellular level. But we must remember that although the theory proposed by Hebb (1949) was called cell assembly theory, he mainly argued that the synapses, their configuration, and cooperation play an important role in learning and other processes, known as “Hebbian Learning”. He also assumed that a neuron can belong to different cell assemblies (Hebb, 1949). Buzsáki (2010), for instance, also talked about *“synapsembles”* as a constellation of current synaptic weights and as the constituents of the neural syntax. However, he admits: “Despite the expected critical role of synapsembles in neural syntax, experimental evidence supporting the role of synapsembles in combining and separating neuronal assemblies is scarce, largely because of the lack of tools to directly measure synaptic connectivity in the behaving animal” (Buzsáki, 2010, p. 372).

To test the hyper-brain cell assembly hypothesis on the *cellular level*, comprehensive hyperscanning studies on animals are needed. The primary goal of such studies should be to test whether neuronal elements fire synchronously in two or more brains during an interaction. The aforementioned studies in mice (Kingsbury et al., 2019) and bats (Zhang and Yartsev, 2019) provide very important information about neuronal cell activity during different interaction situations but are based on correlation data over time. Information about the spatiotemporal patterns of spiking behavior in hyper-brain assemblies is necessary to provide a better understanding of these phenomena. The study of spiking behavior in animals is also important in relation to the configuration and cooperation of synapses in multi-brain activity indicated above. It would be important to investigate how similar or dissimilar these synapse configurations are in the interacting brains. Moreover, there is evidence that molecular manipulations that cause an increase or decrease in the synaptic efficacy in dmPFC neurons in mice can trigger an upward or downward movement in social rank, respectively (Wang et al., 2011). The authors also found that dominance ranking in mice is transitive, relatively stable, and highly correlates with multiple features of dominance behaviors (e.g., aggressiveness, stress responsiveness, fearfulness, etc.). Therefore, such molecular or synaptic manipulations could be effective in combining and separating neuronal assemblies to understand how the behavioral specificity of these assemblies is generated by distinct synaptic weights and their configurations.

On the *brain oscillation level*, at least two techniques are currently attracting attention: multibrain stimulation (MBS) and multibrain neurofeedback (MBN). Both these methods allow the researcher to influence interacting brains in a hyperscanning experiment and change rhythmic activity in a predicted or hypothesized manner. Interestingly, these ideas have already received confirmation in animal studies. It has been shown that imposed interbrain synchrony shapes social interaction and social preference in mice (Kingsbury et al., 2019; Yang et al., 2021). Studies with MBS applications are currently still scarce and the results are inconsistent. Only a few studies have used transcranial alternating current stimulation in a hyperscanning setup (so-called hyper-tACS; see Szymanski et al., 2017a). In a dual finger-tapping task, it has been shown that the pairs improve their performance when their motor cortices are stimulated with beta band (20 Hz) in-phase currents (Novembre et al., 2017). In another hyper-tACS or MBS study, music instructor-learner dyads exhibited spontaneous and synchronized body movement and enhanced learning performance when stimulated with 6-Hz in-phase alternating currents (Pan et al., 2021). Remarkably, these effects were both phase- and frequency-specific: 6 Hz anti-phase stimulation or 10 Hz in-phase stimulation did not produce comparable results. In a study with synchronous dyadic drumming, contrary to the researchers’ expectations, both the same-phase-same-frequency (6 Hz) and the different-phase-different frequency (5 Hz with 13 degrees offset in one participant and 7 Hz with 1 degree offset in the other) conditions were associated with greater dyadic drumming *asynchrony* relative to the sham (no brain stimulation) condition (Szymanski et al., 2017a). In an MBN study (Müller et al., 2021b), neurofeedback was provided either as two balls approaching each other (ball design), or as two pendula, each reflecting the oscillatory activity of one of the two participants (pendulum design). The delta (2.5 Hz) and theta (5 Hz) frequency oscillations were used as neurofeedback features. The participants proved able to increase inter-brain synchrony by using neurofeedback, especially when it was fed back at the theta frequency. Moreover, other oscillatory activities (e.g., power spectral density, peak amplitude, and peak frequency) also changed during the neurofeedback task compared with the rest. Importantly, all the measures showed specific correlations with the subjective post-survey item scores, reflecting subjective feeling and appraisal (Müller et al., 2021b). The disadvantage of the MBS studies presented above is that the brain stimulation took place either in-phase or anti-phase, whereas for the testing of the hyper-brain cell assembly hypothesis it would be essential to shift the phase only slightly in one subject relative to the other. In this way, it might be possible to test whether absolute or exact in-phase synchronization is important for social interaction and for the hyper-brain cell assembly to synchronously ignite cell assemblies in both brains and to integrate them. In the study by Szymanski et al. (2017a), such a phase shift was used, but the stimulation occurred at different frequencies (i.e., 5 and 7 Hz). Furthermore, hyper-tACS have so far only addressed a limited number of homologous brain regions (cf. Novembre and Iannetti, 2021) and all three aforementioned studies used different cortical sites and hemispheres for brain stimulation. Using heterologous stimulation electrodes would be more appropriate for the investigation of inter-brain synchronization during naturalistic social interactions (cf. Novembre and Iannetti, 2021), because inter-brain synchronization mostly concerns heterologous recording electrodes in two brains (e.g., frontal to central, frontal to parietal, etc.). The further disadvantage of the MBS approach is that the stimulation causes severe artifacts in EEG, making a precise examination of brain oscillations during stimulation difficult and sometimes impossible. The MBN approach is much better in this respect, as it does not cause EEG artifacts. Again, one could manipulate the temporal features of the neurofeedback presentation and see how its temporal shift in one subject relative to the other affects hyper-brain neurofeedback performance and the spectral or synchronization indices. As mentioned above, cortical and especially biological systems never operate at only one oscillation frequency. Therefore, it is preferable and more convincing to test the suggested hypothesis with a combination of WFC and CFC, which also involves the multilayer approach. The use of MBS or MBN approaches in different frequencies that have a certain relationship to each other would be particularly interesting, also in terms of phase-to-amplitude CFC or other CFC relationships. Note, however, that all these approaches might have a high potential impact on oscillatory activity modulation during the respective experiment, but such methods can provide only indirect evidence with regard to the suggested hyper-brain cell assembly hypothesis—they cannot substitute for testing the hypothesis at the cellular level as suggested above to examine the respective spiking behavior.

## Concluding Remarks

Synchronization phenomena are ubiquitous and inevitable constituents or emergences of our universe. They are also crucial for our everyday social life, where people often have to coordinate their actions with each other in time and space. It is then also natural and self-evident that these phenomena take place in the brain controlling and mapping our behavior and relationships. Cell assemblies emerging within the interacting brains require a steady adjustment and tight cooperation to justify the interpersonal dynamics and interactive activity that very often operate at ms time scales. In this Hypothesis and Theory article, a *Hyper-Brain Cell Assembly* is hypothesized that encompasses and integrates oscillatory activity within and between brains, and represents a common hyper-brain unit responsible for social and interaction behavior. This hypothesis states that such hyper-brain cell assemblies emerge through joint and simultaneous ignition of neural cells within two or more brains supported by inter-brain synchronization patterns and their ongoing adjustment. Hyper-brain modules or communities comprising nodes across two or several brains and indicating strong relationships between these nodes or brain structures are considered as one of the possible representations of such hypothesized *hyper-brain assemblies*. These assemblies or hyper-brain community structures can also have a multidimensional or multilayer dynamic organization based on WFC and CFC within and between brains or physiological systems and subsystems. It is concluded that the neuronal dynamics during interpersonal interaction ae brain-wide and based on a common neuronal activity of different brain structures within and between brains operating in permanent interaction. Different approaches for testing the hyper-brain cell assembly hypothesis on different levels were proposed. Clearly further sophisticated research is needed to establish our view and deepen our understanding of these highly interesting and complex phenomena.

## Ethics Statement

The studies involving human participants were reviewed and approved by the ethics committee of Max Planck Institute for Human Development (Berlin). The patients/participants provided their written informed consent to participate in this study.

## Author Contributions

The author confirms being the sole contributor of this work and has approved it for publication.

## Conflict of Interest

The author declares that the research was conducted in the absence of any commercial or financial relationships that could be construed as a potential conflict of interest.

## Publisher’s Note

All claims expressed in this article are solely those of the authors and do not necessarily represent those of their affiliated organizations, or those of the publisher, the editors and the reviewers. Any product that may be evaluated in this article, or claim that may be made by its manufacturer, is not guaranteed or endorsed by the publisher.
